# Polypharmacy and Falls in the Elderly: A Literature Review

**DOI:** 10.5812/nms.10709

**Published:** 2013-06-27

**Authors:** Tania Hammond, Anne Wilson

**Affiliations:** 1 Lower Eyre Health Services, Cummins and Districts Memorial Hospital, Australia; 2 School of Medicine, University of New South Wales, Flinders University of South Australia, Bedford Park, Australia

**Keywords:** Polypharmacy, Falls, Older people, Literature review

## Abstract

**Context:**

Medications are taken to ease, control or cure ailments. They are effective and safe if used correctly. In the elderly, disorders that occur as a result of ageing, frequently require treatment, resulting in increased use of medications. Polypharmacy is common among the elderly and although it can be therapeutic in nature, is linked to adverse events such as falls.

**Evidence Acquisition:**

A review of the literature was conducted. English articles in Cinahl, Medline and Healthsource (2000-2012) were searched for links between polypharmacy and falls in older adults aged 65 years old and over. Articles not meeting the age criterion were excluded.

Search terms included falls, polypharmacy, medications, multiple medications, medicines, elderly, aged. A total of 120 articles were retrieved from the Literature search.

**Results:**

Sixteen articles were included in the literature review. Four literature reviews, three observational prospective cohort, three cross-sectional, three case-control, one longitudinal study and two retrospective cohort studies were examined. Many studies were able to demonstrate a link between the number of medications taken and risk of falls however the potential for bias resulting from confounding by indication was high due to study design in many cases.

**Conclusions:**

Polypharmacy as an independent variable has been linked to falls in older people, however there appears to be a stronger link between falls and the type of medications taken (e.g. medications known to increase risk of falls), rather than polypharmacy on its own. Polypharmacy can sometimes be therapeutic and it may be more beneficial to consider terms such as ‘inappropriate prescribing’ or potentially inappropriate medications’ when considering the effects of medication on falls in older adults. Polypharmacy in older people is often viewed in a negative light due to the increased risk of adverse events, including falls. This article examined current knowledge on the characteristics that define polypharmacy, its effect on falls in elderly people and provided recommendations for future research. Further research utilizing prospective and intervention studies are needed to clarify the causal relationship between polypharmacy, comorbidities and fall risk.

## 1. Context

In any two-week period, nine out of ten elderly Australians take at least one medication (1). The presence of co-morbidities, age-related physiological changes and an ageing population means that older people are frequently prescribed a high number of medications, often referred to as polypharmacy (2).

Polypharmacy is linked to increased risk of adverse drug events in older people due to increased risk of drug interactions, lack of adherence to medication regimes, susceptibility of older people to side effects of medications, and physical changes related to ageing causing difficulties in taking medications as intended (2, 3).

In Australia, adverse drug events are responsible for more than 30% of unplanned admissions to hospital in elderly people 75 years and over (4). Also, repeated admissions related to adverse drug events have increased at a much greater rate than ‘first-time’ admissions for adverse drug events (5).

One adverse event that can be related to medications and polypharmacy in the elderly is falls. Falls are known to be a serious health problem for older people (6, 7). As the percentage of elderly adults in the Australian population grows, falls in this group have come to the forefront as a serious and growing health concern. Approximately 30% of community-dwelling older people fall each year and the consequences of these falls can be catastrophic, resulting in loss of quality of life, fear of falling, depression and general lack of self-confidence.

Medications are often associated with an increased risk of falls and it is generally accepted that the risk of falls increases with the number of medications taken, with those taking four or more medications at greater risk of falling (8-12). Furthermore, the type of medications or specific medications ingested has been shown to significantly influence the fall risk (7).

Despite an abundance of literature on the subject, there is no universally accepted definition of polypharmacy. It is defined in the literature as the use of a number of medications taken at the same time, the number of medications varies from 2 to 5 or more, depending on the study (8, 10). Some definitions include over the counter and complementary medications, while others consider prescription medications only (8, 10). Some studies use a list of characteristics to define polypharmacy, regardless of the number of medications taken; for example unnecessary or excessive use of medications (13). Brager and Sloand (2) define polypharmacy as the use of two or more medications with the following characteristics:

- To treat the same condition
- Of the same chemical class
- With the same or similar pharmacologic actions to treat different conditions

The authors highlight that polypharmacy should not always be considered in a negative light, and is sometimes an appropriate and therapeutic treatment strategy. Brager & Sloand (2) consider different types of polypharmacy and believe ‘irrational polypharmacy’ to be the type that can have detrimental effects on older people.

Some authors do not use the term polypharmacy at all, instead opting for terms such as ‘inappropriate prescribing’ or ‘potentially inappropriate medications’ to explain the phenomenon of polypharmacy and how it affects older people (3, 14).

Falls in the elderly population are recognized as a leading cause of mortality and morbidity with increased hospitalizations and drain on health systems. This paper reports on published literature examining polypharmacy as a risk factor for falls in the elderly and provides information on how to address the gaps in knowledge.

The purpose of this literature review was to establish published theoretical viewpoints on polypharmacy as a risk factor for falls in the elderly and produce an overview on the subject.

## 2. Evidence Acquisition 

Medline, CINAHL and HEALTHSOURCE databases were searched for original English articles published between January 2000 and September 2012. Search terms included falls, polypharmacy, medications, multiple medications, medicines, elderly, aged.

Cochrane Library reference lists and retrieved articles reference lists were examined for articles not already retrieved. A total of 120 articles were retrieved from the Literature search.

Abstracts of the original 120 articles found through the literature search were read. The following criteria were used to exclude articles:

- Sample aged below 65 years of age (or a mean age of less than 65 years)
- Sample did not include community-dwelling older adults
- Studies/articles relating to specific classes of medications
- Studies/articles that did not clearly demonstrate a link between polypharmacy and falls in older adults
- Articles that discussed falls risk factors, but did not include medications or polypharmacy

Twenty-four articles remained after sorting, based on the above criteria. Eight Of these 24 articles were excluded as they were information-based articles only, not literature reviews or reports of research studies.

The remaining 16 articles were included in this literature review (Table 1).

**Table 1.** Summary of Articles Included in the Literature Review

Author	Study/ArticleType	Number of Participants	Age of Participants, Years	Definition of Polypharmacy	Association with Falls
**Buatois et al. 2010**	Population-based Prospective study	1618	Over 65	Four or more medications	Four or more medications/day a variable that can predict falls
**Fick et al. 2008**	Retrospective, Cohort	17,971	Over 65	Potentially inappropriate medications	Higher incidence of falls, hip and femur fractures than comparison group
**Freeland et al. 2012**	Retrospective Cohort	118	Over 65	Four or more medications	14% increase in fall risk with the addition of each medication beyond a 4 medication regimen
**French et al. 2005**	Case-control	2212	Median age 77	-	Linked to combination of meds known to cause falls
**Gallagher et al. 2007**	Literature review		Over 65	Inappropriate prescribing	Linked to a range of adverse reactions in older adults, including falls
**Ganz et al. 2007**	Literature review	Older people	Older people	-	Links to number and type of medications taken
**Hartikainen et al. 2007**	Systematic literature review	Older people	Older people	Four or more medications	Increased number of medications associated with inc falls
**Kelly et al. 2003**	Case-control	2278	Over 66	-	Increased risk of falls associated with types of medications rather than number
**Kojima et al. 2011**	Cross-sectional study	262	mean age 76.2 ± 6.8	Multiple drug use	Polypharmacy rather than number of comorbidities was associated with fall risk
**Kojima et al. 2012**	longitudinal observational study	172	mean age 76.9 ± 7.0	Multiple drug use	Polypharmacyis associated with falls
**Lawlor et al. 2003**	Cross-sectional study	4050		-	Falls increased with increase in number of medications as with increase in comorbidities
**Tromp et al. 2001**	Prospective cohort study	1285	Over 65	-	Four or more medications increases risk of falls
**Veehoff et al. 2000**	Literature review	Older people	Older people	Two or more medications	Standard of literature poor, need for further research
**Weber et al. 2007**	Prospective study with control group	620	Over 70	Four or more medications	No significant reduction in falls intervention group
**Wilson NM, Hilmer SN, March LM, et al.**	Retrospective study. Data from RCT	602	Mean age was 85.7 ± 6.4,	number of different medications	DBI is significantly and independently associated with falls in older people
**Ziere et al. 2005**	Population-based Cross-sectional study	6928	Median age 70.6	Four or more medications	Risk of falling increases with number of medications used/day

## 3. Results

Of the reviewed sixteen articles there were four literature reviews, three observations of prospective cohorts, three cross-sectional, three case-control, one longitudinal study and two retrospective cohort studies. Sample sizes in relevant studies ranged from 118 (15) to almost 18,000 (14).

The most common definition of polypharmacy used in the studies was ‘the use of four or more medications’ (12, 16-18). Despite making references to polypharmacy, five studies did not include a definition. However, polypharmacy was not the primary theme of these studies (9, 11, 19, 20). All but two papers identified links between polypharmacy and falls. Some studies cited links between increased falls and an increase in the number of medications used per day (12, 15, 17, 20). Other studies concluded that increased falls risk is associated with the type of medication used rather than the number of medications used, although the likelihood of being prescribed a medication that is known to cause falls increases with the number of medications taken (11, 17, 19). One study identified that elderly people taking one or more anticholinergic or sedatives had almost doubled the risk of having a fall over a year compared with people taking less than one of either drugs (7).

French et al. (9) and Kelly et al. (19) used case-control methods to study the relationship between medications and hip fractures and medications and falls, respectively. Case-controls are more accurately able to identify causality between medications and falls as they consider medications taken leading up to, and at the time of the fall. Other study design methods do not allow for this consideration.

Veehoff, Jong & Hajjar-Ruskamp (2000), attempted to find a workable definition of polypharmacy and the implications of the phenomenon in general practice, however they were unable to draw many conclusions due to the lack of quality literature available at the time.

## 4. Conclusion

The review of the literature showed that because there is no universally accepted definition of polypharmacy it is not always considered a risk to the elderly’s well-being. Although there is a large amount of literature available from studies conducted on the subject but, they were not always rigorous, utilizing weak study designs.

Significantly, the review identified links between polypharmacy, and increased falls that was associated either with the type of medications taken or, as in some situations, were linked with an increase in the number of medications used (21) per day. The association of fall risk with comorbidities and medications was confirmed (6). Unfortunately, falls in the elderly can result in hip fractures and subsequent sequelae to reduced quality of life or longevity.

Prior to 2000, polypharmacy was generally considered an independent risk factor for falls in the elderly, however more recent evidence suggests that polypharmacy alone is unlikely to be the risk factor (12, 18). The association between increased falls risk and polypharmacy appears to be much stronger when the older person is taking at least one medication that is known to cause falls (7, 12, 18). Such medications may almost double the risk of having a fall.

A more appropriate way of looking at the effects of polypharmacy on falls in older adults appears to be ‘inappropriate prescribing’ rather than the number of medications taken. This takes into consideration the types of medications prescribed, reasons for prescreption, things to consider when commencing certain types of medication, potential effects on the older person and reasons for omission of certain types of medications (2).

Considering polypharmacy as purely the number of medications taken by an older person can be limiting and adherence issues may not be considered. Studies have shown that polypharmacy can lead to omissions by older people due to the complex nature of their medication regime, which in turn can cause adverse effects. A missed medication can cause hospitalization due to adverse drug events just as much as a medication that has been ingested (5).

Most studies retrieved were observational epidemiological studies, in particular prospective or cross-sectional. The potential for bias in these types of studies, as a result of confounding by indication, is quite high. Interventional and prospective studies designed for the elderly population are needed to determine strategies and guidelines to reduce falls.

This review points out that further research is required to fully understand the phenomenon of polypharmacy and its effect on older people, not only in relation to falls but other adverse effects. A common understanding of what is considered to be polypharmacy is essential and should include characteristics other than simply the number of medications taken. Through the review, the authors have identified that a universal definition would aid research, improve clinical practice by the development of standardized guidelines and address potential risks to the elderly population. Further research utilizing prospective and intervention studies are needed to clarify the causal relationship between polypharmacy, comorbidities and fall risk.
